# Unveiling Tatun volcanic plumbing structure induced by post-collisional extension of Taiwan mountain belt

**DOI:** 10.1038/s41598-021-84763-z

**Published:** 2021-03-05

**Authors:** Hsin-Hua Huang, E.-S. Wu, C.-H. Lin, J. Y.-T. Ko, M.-H. Shih, I. Koulakov

**Affiliations:** 1 Institute of Earth Sciences, Academia Sinica, Taipei, Taiwan; 2 Institute of Oceanography, National Taiwan University, Taipei, Taiwan; 3 Trofimuk Institute of Petroleum Geology and Geophysics SB RAS, Novosibirsk, Russia; 4 Novosibirsk State University, Novosibirsk, Russia; 5 Institute of Volcanology and Seismology FEB RAS, Petropavlovsk-Kamchatsky, Russia

**Keywords:** Seismology, Volcanology

## Abstract

The Tatun Volcanic Group (TVG) is proximal to the metropolis of Taipei City (population of ca. 7 million) and has long been a major concern due to the potential risks from volcanic activity to the population and critical infrastructure. While the TVG has been previously considered a dormant or extinct volcano, recent evidence suggests a much younger age of the last eruption event (~ 6000 years) and possible existence of a magma reservoir beneath the TVG. However, the location, dimension, and detailed geometry of the magma reservoir and plumbing system remains largely unknown. To examine the TVG volcanic plumbing structure in detail, the local P-wave travel time data and the teleseismic waveform data from a new island-wide Formosa Array Project are combined for a 3D tomographic joint inversion. The new model reveals a magma reservoir with a notable P-wave velocity reduction of 19% (ca. ~ 19% melt fraction) at 8–20 km beneath eastern TVG and with possible northward extension to a shallower depth near where active submarine volcanoes that have been detected. Enhanced tomographic images also reveal sporadic magmatic intrusion/underplating in the lower crust of Husehshan Range and northern Taiwan. These findings suggest an active volcanic plumbing system induced by post-collisional extension associated with the collapse of the orogen.

## Introduction

**Figure 1:**
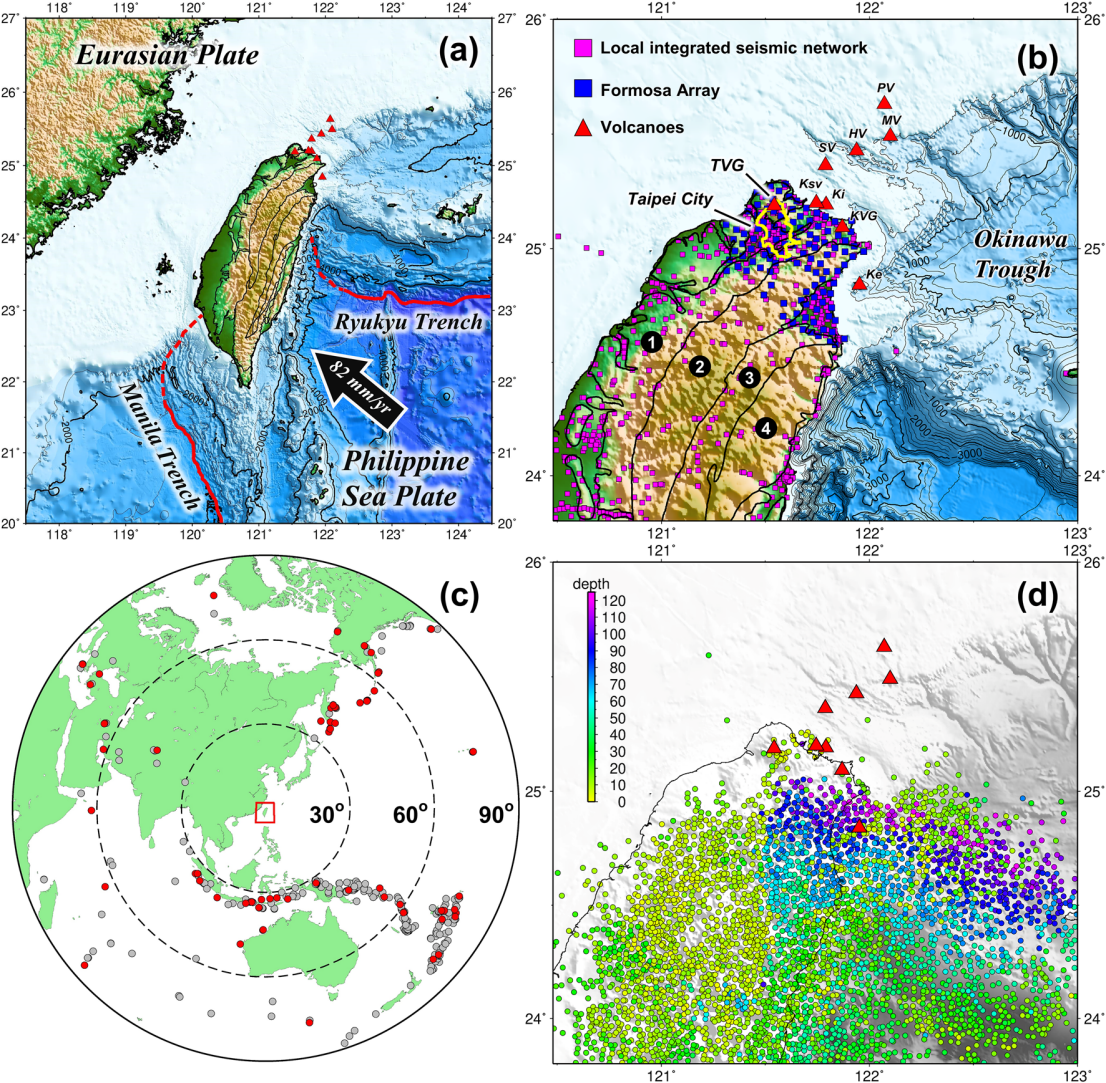
Tectonic and data distribution maps. (**a**) Tectonic setting of the Taiwan region where the Philippine Sea plate subducts northward along the Ryukyu trench and the Eurasian plate subducts eastward along the Manila trench. The black arrow shows the plate motion at 82 mm/year. (**b**) Study area showing the distribution of stations used and regional volcanoes. Purple and blue squares denote the stations of local integrated seismic network and Formosa Array. Red triangles indicate the volcanoes^9^. *TVG* Tatun volcano group, *KVG* Keelung volcano group, *Ke* Kueishantao Island, *Ki* Keelungyu Island, *Ksv* Keelung submarine volcano, *SV* An unnamed submarine volcano, *HV* Huapingyu island, *MV* Mienhuayu island, *PV* Pengchiayu island. Label 1–4 shows geological units of western foothill, Hsuehshan Range, western and eastern Central Range, respectively. Taipei City is outlined by the yellow curves. (**c**) Distribution of teleseismic events recorded (gray dots) and used (red dots) by Formosa Array deployment period. (**d**) Distribution of used local earthquakes color coded by focal depth. This figure is generated by GMT 4.5.18 https://www.generic-mapping-tools.org/.

The Taiwan orogenic belt is a product of oblique collision between the Eurasian plate (EP) and the Philippine Sea plate (PSP), where the EP subducts eastward at the Manila trench to the south and the PSP subducts northward at the Ryukyu trench to the east (Fig. 1a). The collision initiated 4–6 Ma as the PSP Luzon arc collides toward the EP continental margin and propagated southward from northern Taiwan^1,2^. The northern Taiwan has therefore experienced a tectonic transition from collision to post-collisional extension since ca. 2.8 Ma^3–5^ producing multiple-stage and complex volcanisms^6,7^ (Fig. 1b). Offshore in the east, the PSP subduction and subsequent back-arc opening created the Ryukyu volcanic arc and a series of submarine volcanoes in the Okinawa trough and Kueishantao Island (Ke). Offshore in the north, the northern Taiwan volcanic zone (NTVZ) in NE-SW trending was suggested to form by the extensional collapse of the mountain belt^3,6^. The most obvious inland manifestation of this NTVZ is the Tatun volcano group (TVG) located north of Taipei city. Its proximity within 15 km of the metropolis Taipei where more than 7 million people reside and two nuclear power plants on the northern coast has posed a severe threat to communities across Taiwan^8^.

The TVG has been considered an dormant or extinct volcano until recently since no volcanic eruption was recorded in historical times^8,10^. The two major time periods of volcanism pulses were suggested at 2.8–2.5 Ma and 0.8–0.2 Ma by the radiometric dating of volcanic rocks^11–13^. However, recent studies of volcanic ash characters in the northern Taipei basin and volcaniclastic deposits in the TVG found much younger eruption records of 20 Ka and 6000 years ago^14,15^. Meanwhile, growing evidences also suggested a magma reservoir likely existing beneath the TVG. For instance, the high ^3^He/^4^He ratio from the fumarolic and bubbling gasses of TVG hot springs requires a deep magmatic origin, e.g. magma reservoir^16,17^. The seismic analysis of S-wave shadows and P-wave delays from deep subduction earthquakes suggested the existence of a magma reservoir in the lower crust slightly to the east of the TVG^18^. While counterevidence for such a lower-crust magma reservoir was also proposed recently^19^, the vigor of hydrothermal activity at several fumarole sites, identified volcanic tremors and phreatic eruptions, and frequent miscroearthquake activities^8,20–22^ all require a persistent heat source beneath the TVG. It is therefore of great importance to image the location, dimension, and detailed geometry of the feeding system.

To illuminate the volcanic plumbing structure of the TVG, a deployment of dense broadband seismic array, named Formosa Array^23^, was launched in 2017 and has installed 120 out of 140 planned stations by October 2019 (Fig. 1b, blue squares). The station spacing is about 5 km uniformly across plain and mountain areas, providing a new opportunity to improve tomographic imaging of northern Taiwan^24–27^. In this study, we combine the teleseismic waveform data from the Formosa Array and the local P-wave picking data from an integrated seismic network to conduct a 3D joint inversion (Fig. 1c,d). The new model unveils a clear magma reservoir beneath the east of the TVG and possible magmatic intrusion/underplating bodies related to post-collisional extension in northern Taiwan.

## Results

### Model results and verification

**Figure 2:**
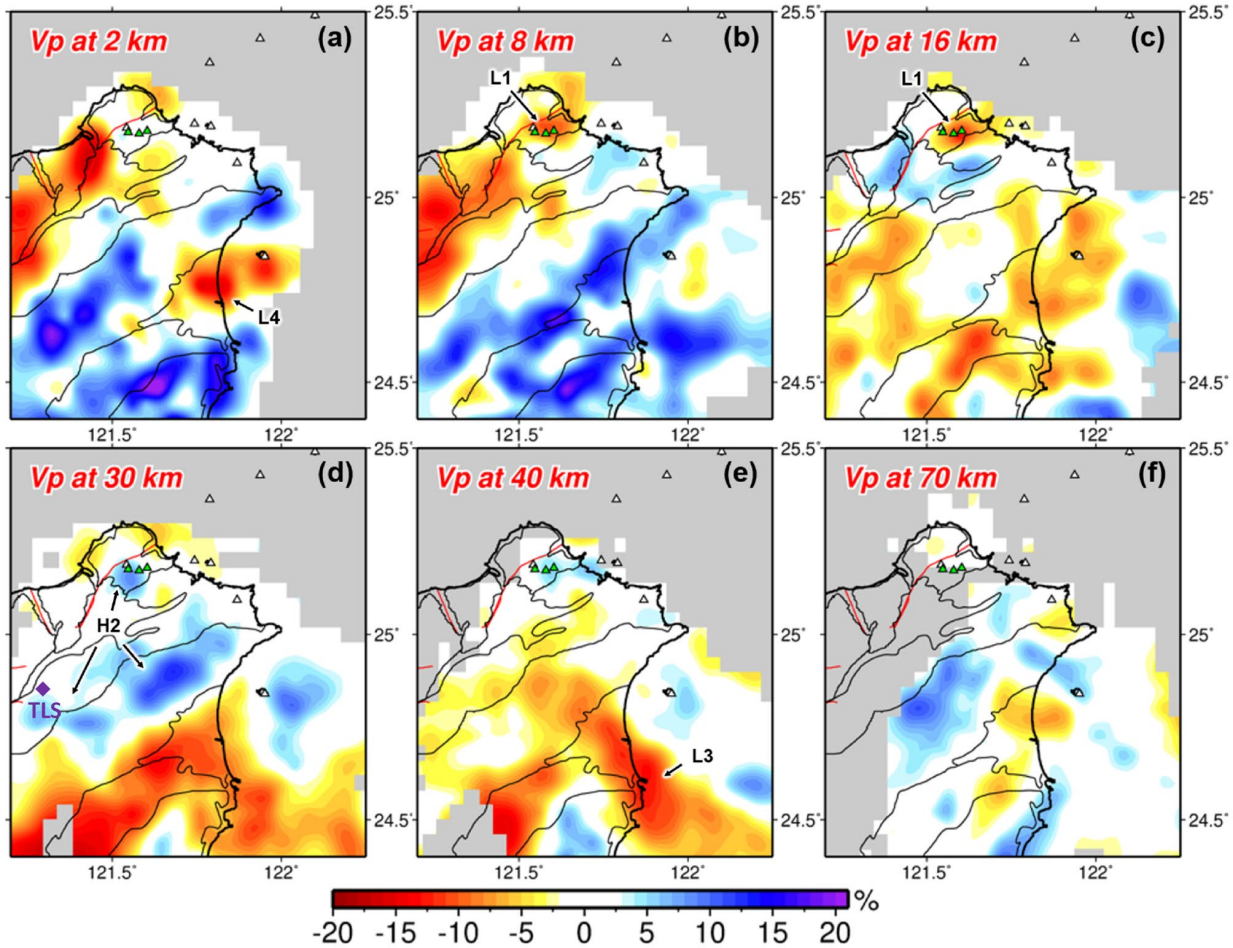
Model results at different depths (**a**–**f**). The warm and cold colors represent the low and high P-wave velocities. White triangles denote the volcano locations of Northern Taiwan volcanic zone. Green triangles are the main fumarole sites and craters in Tatun volcano group. The purple diamond in (**d**) shows the Tsaolingshan lava site (TLS). Labels indicate the velocity anomalies discussed in the text. This figure is generated by GMT 4.5.18 https://www.generic-mapping-tools.org/ and Microsoft Office 2016 https://www.microsoft.com/zh-tw/microsoft-365/microsoft-office?rtc=1.

**Figure 3:**
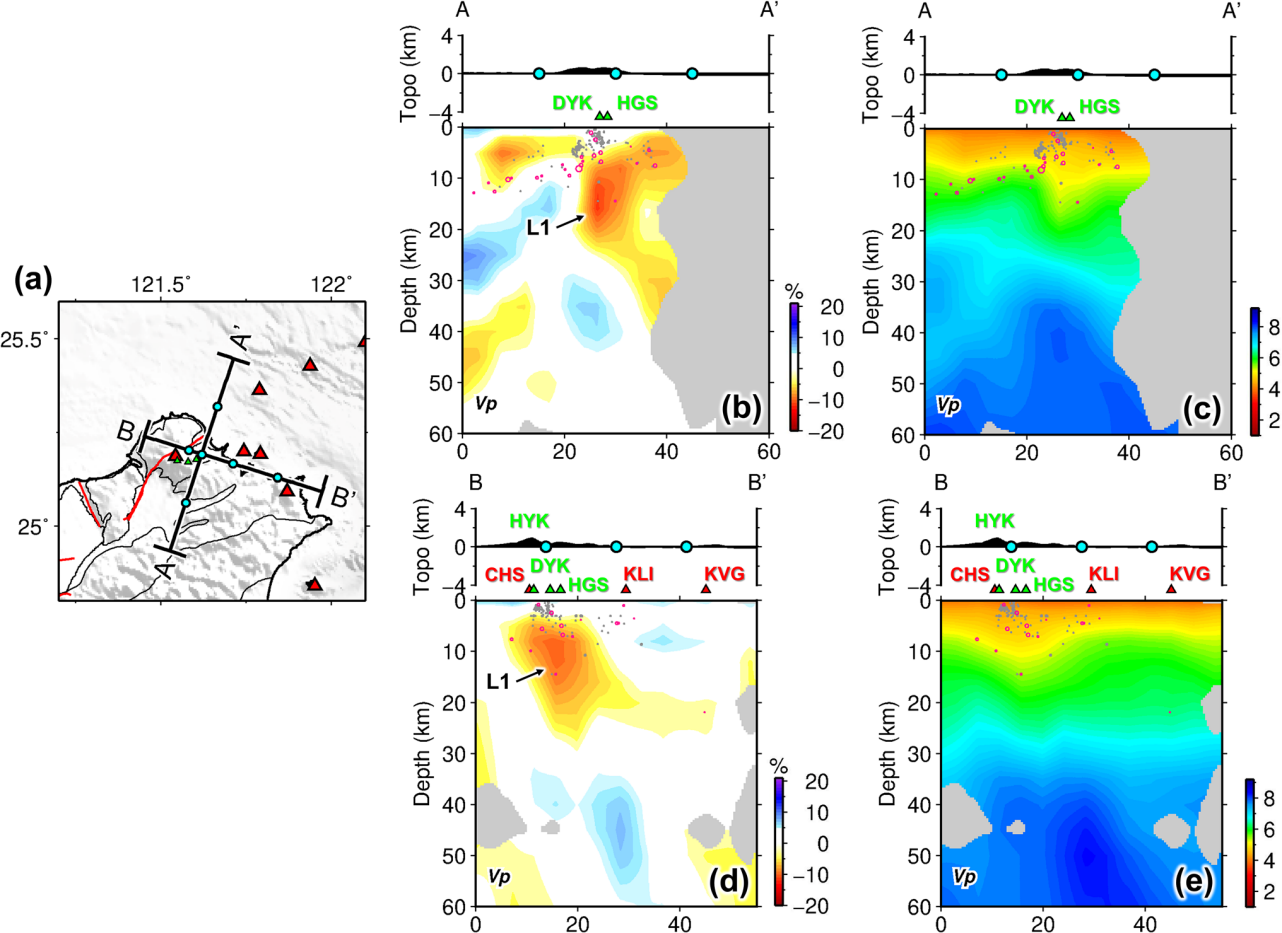
Model results in cross sections across the Tatun volcano group. (**a**) Locations of cross sections AA′ and BB′ shown in (**b**–**e**). Cyan dots mark the distance intervals of cross-sections for reference. P-wave velocity perturbations and absolute values for cross-sections AA′ and BB′ are shown in (**b**,**d**) and (**c**,**e**) respectively. Red triangles denote the volcano locations of Northern Taiwan volcanic zone. Green triangles are the main fumarole sites and craters in Tatun volcano group. The pink and gray dots denote earthquakes from two different catalogs: the former are the groping events used in the inversion and the latter are local microseismicity determined by TVO seismic network. Labels indicate the velocity anomalies discussed in the text. This figure is generated by GMT 4.5.18 https://www.generic-mapping-tools.org/ and Microsoft Office 2016 https://www.microsoft.com/zh-tw/microsoft-365/microsoft-office?rtc=1.

The model slices at different depths are shown in Fig. 2, where poorly resolved areas are masked and defined as a resolvability index R < 0.6 calculated from checkerboard tests (Supplementary information and Supplementary Fig. S5). Benefiting from the uniform and dense station distribution of the Formosa Array, the joint inversion integrating teleseismic data greatly improves the resolution to the north of latitude 25.3° and at depths of 5–50 km when compared to the inversion results without teleseismic data (Supplementary Figs. S1 and S6). With the enhanced images, a pronounced low velocity anomaly (L1) stands out beneath the Tatun volcano group (TVG) at the depth slice of 8 and 16 km (Fig. 2b,c). This slow anomaly is elongated sub-vertically at a depth range of 8–20 km as shown in cross-sections AA′ and BB′ (Fig. 3b,d), with most of the seismicity lying above the anomaly. According to the projected locations of craters (green triangles in Fig. 3), this slow anomaly is not located at the center of TVG (i.e. Chihsingshan, CHS) but slightly to the east beneath the Huangzuishan (HGS) and Dayoukeng fumarole (DYK). In the cross-section AA′, it can be seen that the reservoir extends upward to a shallower depth of ~ 5 km and northward to the offshore areas near where active submarine volcanoes (SV and Ksv in Fig. 1b) have been documented^9^.

**Figure 4:**
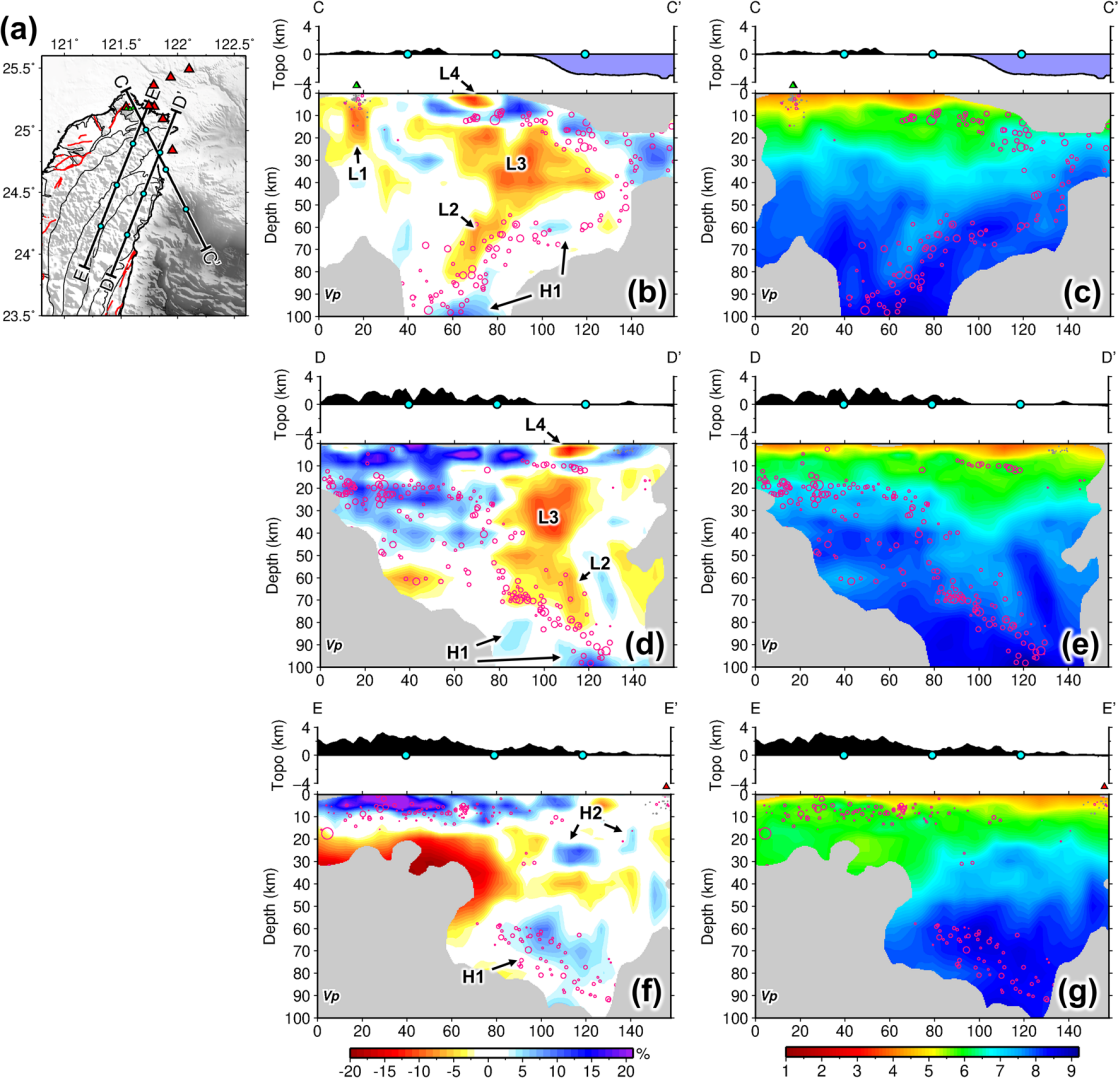
Model results in regional cross sections of northern Taiwan. (**a**) Locations of cross sections CC′–EE′ shown in (**b**–**g**). Cyan dots mark the distance intervals of cross-sections for reference. P-wave velocity perturbations and absolute values for cross-sections CC′–EE′ are shown in (**b**,**d**,**f**) and (**c**,**e**,**g**) respectively. Purple circles indicate the local earthquakes. Red triangles denote the volcano locations of Northern Taiwan volcanic zone. Green triangles are the main fumarole sites and craters in Tatun volcano group. Labels indicate the velocity anomalies discussed in the text. This figure is generated by GMT 4.5.18 https://www.generic-mapping-tools.org/ and Microsoft Office 2016 https://www.microsoft.com/zh-tw/microsoft-365/microsoft-office?rtc=1.

Deep velocity structures beneath northern Taiwan are shown in regional cross-sections in Fig. 4. In cross-section CC′, the subducting PSP is characterized by the seismicity with high velocity anomaly, H1 (Fig. 4b,c). The low velocity anomaly L2 at a deep depth around 70–80 km has been identified and suggested as the subduction-induced partial melting in the previous studies^23,25^. This slow anomaly L2 seems not to be connected to the Tatun slow anomaly (L1) as a feeding source. Other two pronounced low velocity anomalies, L3 and L4, at shallower depths have been observed previously and inferred as the serpentinized mantle wedge^25,27^ and sediments of Ilan plain^25^, respectively. Cross-section DD′ provides another angle of view for these structures (Fig. 4d,e). Further to the west in cross-section EE’, it is noteworthy that an abrupt transition in crustal structure is observed from thickened crustal root in the south to a few high velocity anomalies, denoted as H2, in the north (Fig. 4f,g). This transition occurs roughly along the boundary between the Central range and Hsuehshan range (Fig. 2d).

Characteristic-model tests are conducted to evaluate the robustness of the geometry of the TVG L1 slow anomaly (Supplementary Fig. S7). We test vertically-elongated and concentrated synthetic slow anomalies at depth ranges of 8–20 and 12–16 km and find that the vertically-elongated shape of L1 is resolvable and not a consequence of smearing effect. Other tests including data noise and different initial models are also conducted: adding random noise with 0.12-s standard deviation (based on the residual RMS of actual inversion) to the synthetic data and using a different initial model (e.g. 1-D velocity model) do not change the tomographic imaging that much (Supplementary Figs. S8 and S9). Detailed descriptions of the tests are referred to the Supplementary Information.

## Discussion

### Magma reservoir of Tatun volcano

The new model derived from joint inversion of local earthquake and teleseismic data unveils a pronounced low velocity anomaly (L1) beneath the TVG, interpreted as the magma reservoir (Figs. 2, 3). It is vertically elongated in shape and is about 10 km in diameter at depths of 8–20 km, which is shallower than the previously proposed depth^18,19^. This vertically extended geometry of the imaged reservoir seems to be robust through characteristic-model tests (Supplementary Fig. S7–S9). The maximum P-wave velocity (Vp) reduction of the reservoir is about 12%. Since the regularization of tomographic inversions often damps the magnitude of velocity perturbations, we conduct synthetic tests with different input Vp reductions for the reservoir to assess the actual velocity anomaly magnitude (Supplementary Information and Supplementary Fig. S10). The results find that a greater Vp reduction of 19% is needed to fit the inversion results (Supplementary Fig. S10a,f). This is a large value comparable to recently reported high-melt-reservoirs at Long Valley caldera in USA^28^ and Santorini volcano in Greece^29^.

**Figure 5:**
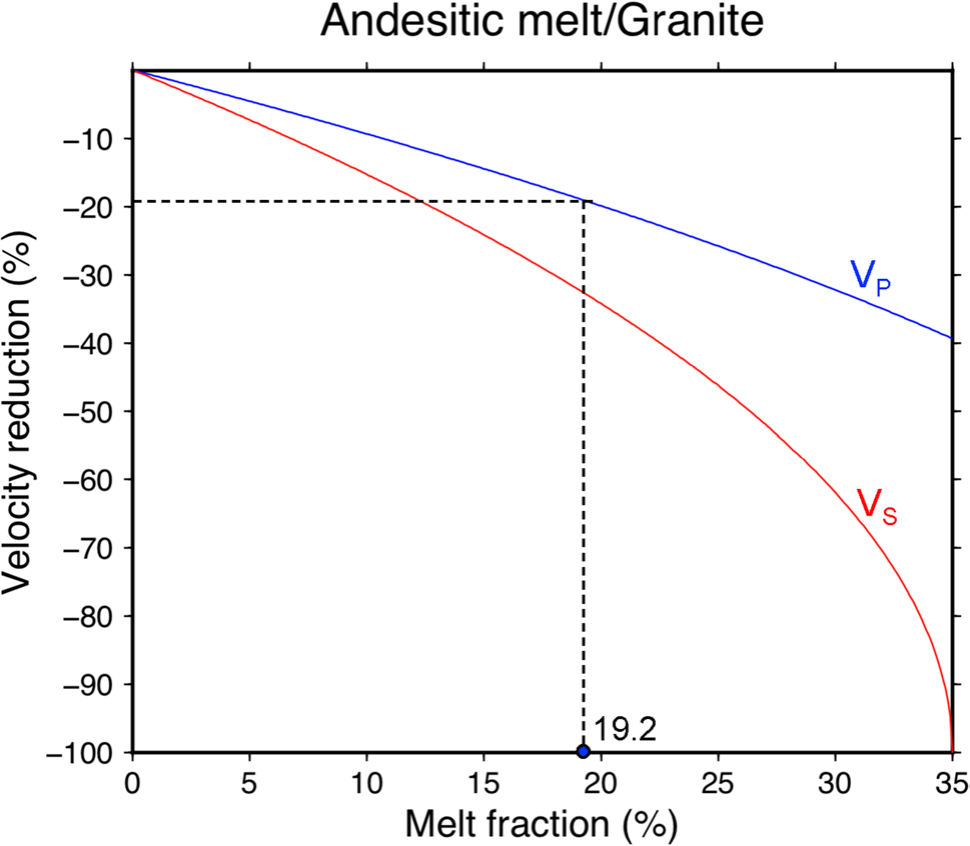
Melt fraction estimation for the magma reservoir. Blue and red curves are calculated relationship of P-wave and S-wave velocity reduction as a function of melt fraction.

The high value of 19% is difficult to explain by temperature and composition alone and implies the presence of melt. We estimate the melt fraction of the magma reservoir by using Gassmann’s relations^30,31^. The calculation and used parameters are described in “Data and methods” and Supplementary Table S2 in detail. Assuming andesitic melt and granite frame rock, 19% Vp reduction yields a high melt fraction of ~ 19% (Fig. 5). Alternatively, replacing andesitic melt by water or CO_2_ for a hydrothermal reservoir results in a pore fraction of ~ 15% (Supplementary Table S2). While it is unlikely to have hydrothermal systems down to as deep as 20 km due to high pressure, a certain mixture such like partial melt intruded at depths and hydrothermal gas/fluid accumulating at the top is possible. Although the uncertainty of this simplified estimation is not trivial, the high melt fraction indicates that the TVG plumbing system could remain active with sufficient heat supply.

Pu et al.^32^ recently relocated the micro-earthquakes in the TVG area and revealed a clear conduit-like structure beneath the Dayoukeng fumarole, which requires volcanic gas and fluid source ascending from below. The consistent location of the magma reservoir we resolved can potentially be the gas/fluid source to either replenish a shallower hydrothermal reservoir or feed directly to the fumaroles through fractured pathways (Fig. 3b).

### Origin of Tatun volcano magma reservoir

In a regional volcanism context, the next key piece of information is to know the origin of magma in the reservoir. The TVG is located above the edge of the northward subducting Philippine Sea Plate (Fig. 1a). But from Fig. 4, it is clear that the magma reservoir (L1) is not fed by current subduction-induced partial melting (L2) as no pathway is identifiable in between. The magmatism of the TVG, as well as the entire northern Taiwan volcanic zone (NTVZ, Fig. 1b), has been suggested to be part of the Ryukyu volcanic front that was initiated by the westward advance of Philippine Sea Plate before 2 Ma and ceased by the following slab rollback and opening of the Okinawa Trough^3,4^. Wang et al., in contrast, suggested a mechanism of post-collisional delamination to form the NTVZ since geochemical characteristics of NTVZ magmas show significant components of asthenosphere and metasomatized subcontinental mantle^4,6^. If extensional magmatism occurred, we envisage that the crust should have been subjected to extensive magmatic intrusion or underplating which is often characterized by high velocity bodies in the lower crust or beneath Moho^33^. In Figs. 2d and 4f, we do observe such plausible high velocity bodies (denoted as H2) sporadically present in the lower crust of the Hsuehshan range and its north according to the Moho depth around 30 km in this region^34–36^. To the south, the crust is thickened and relatively intact along the Central Range (Fig. 4f,g). Distribution of these faster anomalies also largely overlays with the extent of NTVZ volcanism including the Tsaolingshan lava site (TLS) at the southern end of NTVZ^7^ (Fig. 2d, purple diamond), as strong evidence of the magmatic intrusion/underplating during the post-collisional extension of northern Taiwan mountain belt which is still ongoing to the present day^5,37^.

**Figure 6:**
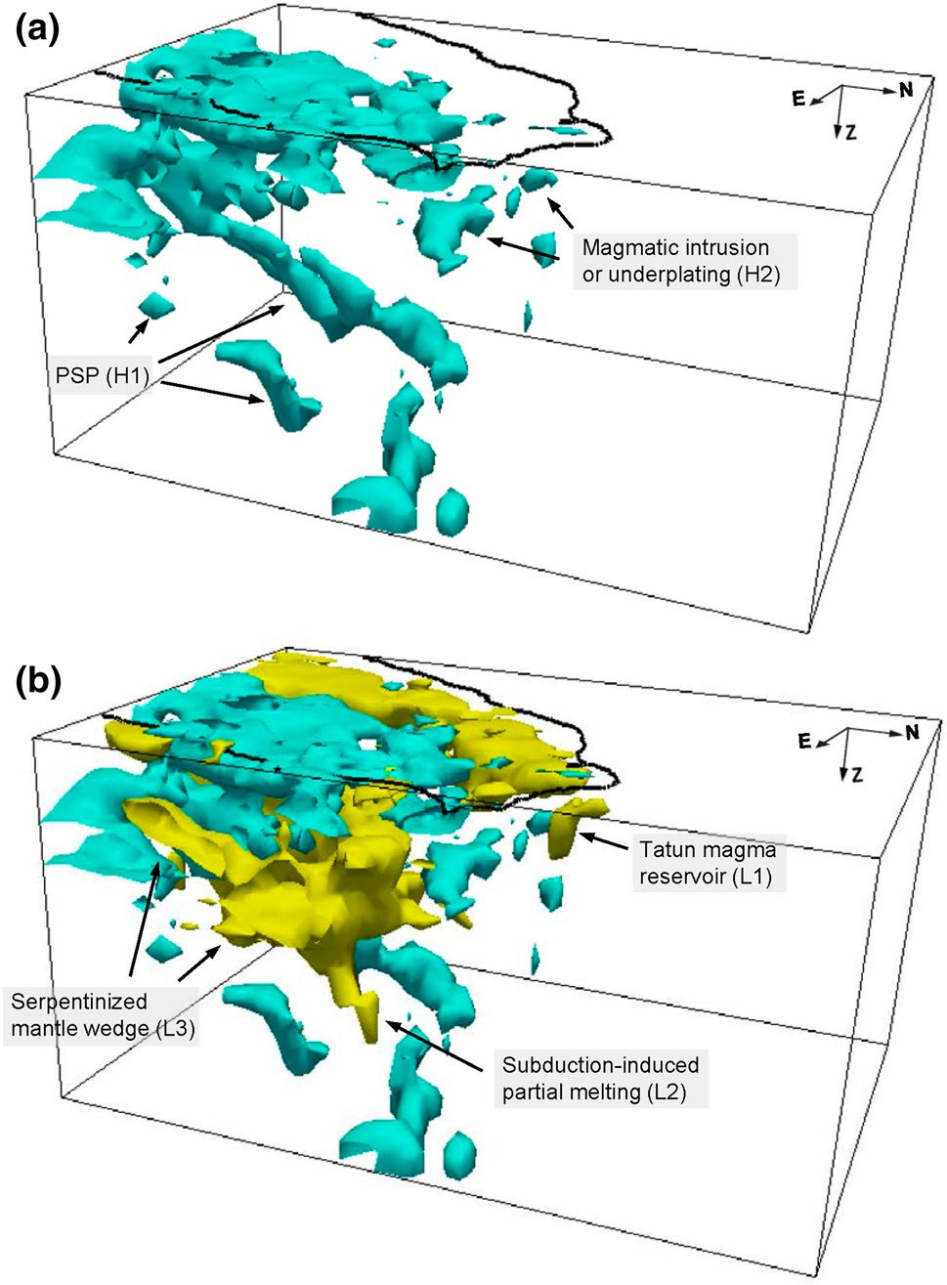
3-D perspective and interpretations of 3-D velocity structures in the northern Taiwan. Yellow and cyan bodies represent the low and high velocity anomalies with values greater than ± 6%.

A clearer 3-D perspective view of northern Taiwan velocity structure is shown in Fig. 6. In addition to features, such as subduction-induced partial melting, serpentinized mantle wedge, and thickened crustal root, the new model unveils the location and geometry of the Tatun magma reservoir and magmatic intrusion/underplating bodies in the lower crust of northern Taiwan, which sheds light on a clearer picture of post-collisional magmatism and tectonics of Taiwan orogeny. In fact, during the slab roll back of Teng’s model^3,4^, extensional magmatism could also occur^38^. By current tomographic imaging solely, it could be difficult to differentiate the slab roll back^3,4^ and the delamination model^4,6^. A higher-resolution imaging of slab interaction between the PSP and EP at deep depths beneath the northern Taiwan may help resolve the debate. However, limited by the relatively narrow aperture of Formosa array, the teleseismic data do not provide additional resolution deeper than 60–70 km (Supplementary Fig. S5).

## Concluding remarks

The 120 uniformly-distributed broadband stations of the Formosa Array provide a unique opportunity to illuminate the Tatun volcanic plumbing system and deep subsurface velocity structure beneath northern Taiwan (Fig. 1b). The wealth of this new dataset will facilitate further studies and findings to come. The unveiled Tatun magma reservoir shows a shallower location (8–20 km) than previously thought and high melt content (~ 19%). Since no clear mantle feeding source is identified, whether the melt is residual from a recent eruption or is new from the mantle source is unknown. While the high melt does not imply an imminent eruption, it could probably sustain an active hydrothermal system of the Tatun volcano in the long run. Monitoring all forms of activity in the TVG (e.g. hydrothermal, microseismicity, gass/fluid chemistry, CO_2_/SiO_2_ flux, ground deformation, etc.) is therefore of great importance for volcanic hazard forecasting and mitigation.

## Data and methods

### Seismic networks and data processing

We use both local and teleseismic earthquake data for a joint inversion in this study. The local earthquake data are compiled from an integrated regional seismic network that combines (1) the Central Weather Bureau permanent seismic network (CWBSN)^39^, (2) Taiwan Strong Motion Instrumentation Program (TSMIP)^40^, (3) Broadband Array in Taiwan for Seismology (BATS)^41^, (4) stations from the Japan Meteorological Agency (JMA) on the western Ryukyu arc^42^, and (5) stations from the TAIGER project that include inland broadband stations and western offshore OBS arrays^43^. A total of 671 stations are used in the study area (Fig. 1b, purple squares). Based on the data criteria that (1) each event was recorded by at least 4 stations, (2) gap angle of recorded stations less than 180°, (3) focal depth shallower than 120 km, and (4) picking quality less than level 2 (according to the CWB picking criteria), 60,081 events are sorted out from 1991 to 2017. To avoid the dominance from compactly-distributed earthquake clusters for better conditioning the inversion, a 3-D event grouping method^25^ with 2 km radius is applied to homogenize the event distribution and reduce the event number to 3587 with 37,373 readings in the end (Fig. 1d).

For the teleseismic data, we collect waveform data from 371 teleseismic events (M_L_ ≥ 5.0) in a distance range of 30°–90° recorded by the newly-deployed Formosa Array (Academia Sinica, Institute of Earth Sciences, 2017) from April 2018 to October 2019 (Fig. 1c, gray dots). The vertical component of waveform data is bandpass-filtered in 0.08–0.15 Hz to measure the P-wave relative travel times between stations using the adaptive stacking method^43^. Through the iterative measuring process, only those with signal-to-noise ratio larger than 5 and cross-correlation coefficients (CC) greater than 0.9 are retained. After final visual inspection and avoiding dominance in the inversion by the events in Tonga subduction zone from the southeast, we sort and even the events in four quadrants and obtain a total of 70 quality events with 4891 readings (Fig. 1c, red dots). Supplementary Fig. S2 shows the measured relative P-wave travel times for four example events coming from different quadrants. The travel times for the stations around the TVG are consistently delayed regardless of the event azimuth, implying the existence of low velocity anomalies underneath. 3-D distribution of ray paths from the local and teleseismic events are shown in Supplementary Fig. S1.

### Tomographic joint inversion and resolution tests

A recently developed tomographic code for multi-dataset joint inversion is employed in this study^25,26,31^, in which the absolute travel-time residuals from local earthquake data and the relative travel-time residuals from teleseismic data are simultaneously minimized (Supplementary Information). The model grids are parameterized with 4 km in longitude and latitude and increasing intervals from − 5 to 120 km in depth as shown in Supplementary Fig. S1a and Supplementary Table S1. The 3-D P-wave velocity (V_P_) model of Huang et al.^25^ is interpolated to the current model grids and used as the initial model for joint inversion. The damping and smoothing factors are chosen to be 20 and 10 after a series of trade-off tests (Supplementary Fig. S3).

We iterate the inversion until the root-mean-square of travel-time residuals (RMS) reduces insignificantly. After 3 iterations, the RMS is reduced from 0.29 to 0.18 s (~ 40% reduction). The relatively less RMS reduction is because of the 3-D initial model used. The residual distribution for local earthquake and teleseismic data is plotted separately in Supplementary Fig. S4. Both the residuals are effectively reduced and concentrated toward zero after the inversion.

The checkerboard test is conducted for assessing general resolution power of the obtained velocity model (Supplementary Information). In Supplementary Fig. S5, the results of the checkerboard tests show good recovery in most inland region and to the east of longitude 122.1° E as well as above 30 km depth. Below 30 km, the well-recovered region is gradually shifted eastward as the depth increases. At 90 km depth, the well-recovered region is primarily limited beneath the Ilan plan and its east offshore. The recovered checkerboard model is then used to calculate a resolvability index, R, at each model node (Supplementary Equation S5 and Supplementary Fig. S5). R ranges from 0 to 1 where the value 1, 0.5, and 0 indicate the node is 100%, 0%, and − 100% recovered, respectively. R = 0.6 is used as a lower bound for the resolvable nodes in tomographic images^31^.

### Estimation on melt fraction and volume of magma reservoir

We calculate effective elastic moduli of fluid-saturated porous materials using Gassmann’s relations^44^ as

1 $$\frac{{K_{eff} }}{{K_{o} - K_{eff} }} = \frac{{K_{d} }}{{K_{o} - K_{d} }} + \frac{{K_{f} }}{{\varphi \left( {K_{o} - K_{f} } \right)}} , \; \mu_{eff} = \mu_{d} ,$$

where $K_{o}$, $K_{d}$ and $K_{f}$ represent bulk modulus for the material of the frame rock, the drained matrix, and the saturating fluid, respectively. $\mu_{d}$ is shear modulus of the drained matrix. $\varphi$ is porosity. For porosity below the critical value ($\varphi_{c}$) that defines the transition from a medium which is supported by the frame to a medium where solid materials are suspended in the fluid^45^,

2 $$K_{d} = K_{o} \left( {1 - \frac{\varphi }{{\varphi_{c} }}} \right) , \quad \mu_{d} = \mu_{o} \left( {1 - \frac{\varphi }{{\varphi_{c} }}} \right) .$$

Substituting Eq. (2) into Eq. (1), the density and seismic velocities of the fluid-saturated porous material can be obtained by

3 $$\rho_{eff} = \left( {1 - \varphi } \right)\rho_{o} + \varphi \rho_{f} , V_{P} = \sqrt {\frac{{K_{eff} + \frac{4}{3}\mu_{eff} }}{{\rho_{eff} }}} , V_{S} = \sqrt {\frac{{\mu_{eff} }}{{\rho_{eff} }}} ,$$

where $\rho_{o}$ and $\rho_{f}$ are density for the drained matrix and the saturating fluid. Given known density and seismic velocities of frame rocks, Eq. (3) can also be used for deriving elastic moduli $K_{o}$ and $\mu_{o}$. Supplementary Table S2 collects the physical parameters of rocks and fluid for calculation, where we assume the frame rock of granite and saturating fluid of andesitic melt for imaged magma reservoir (L1, Fig. 3). The calculation then yields a relationship of the V_p_ reduction and porosity (i.e. melt fraction) as shown in Fig. 5. So, given the V_p_ reduction of 19% for the magma reservoir from characteristic-model tests in Supplementary Fig. S10, the melt fraction is estimated to be ~ 19%.

## Supplementary Information

Supplementary Information.
