# Supplementary material for: Unveiling Tatun volcanic plumbing structure induced by post-collisional extension of Taiwan mountain belt
Source: Sci Rep. 2021 Mar 5;11:5286. doi: 10.1038/s41598-021-84763-z (PMC7970878; doi:10.1038/s41598-021-84763-z)
Supplement: Supplementary file 1 — Supplementary Information. [file 41598_2021_84763_MOESM1_ESM.pdf]

Supplementary materials to

Unveiling Tatun volcanic plumbing structure induced by post-collisional extension of Taiwan mountain belt

H.-H. Huang[1], E.-S. Wu[1], C.-H. Lin[1], J. Y.-T. Ko[2], M.-H. Shih[1], and I. Koulakov[3,4,5]

1 Institute of Earth Sciences, Academia Sinica, Taipei, Taiwan.

2 Institute of Oceanography, National Taiwan University, Taiwan.

3 Trofimuk Institute of Petroleum Geology and Geophysics SB RAS, Novosibirsk, Russia.

4 Novosibirsk State University, Novosibirsk, Russia.

5 Institute of Volcanology and Seismology FEB RAS, Petropavlovsk-Kamchatsky, Russia

*Corresponding author: Hsin-Hua Huang (hhhuang@earth.sinica.edu.tw)

1. Joint inversion scheme and trade-off tests

A recently developed joint inversion code integrating the local earthquake and teleseismic data is employed in this study[23,24,29]. For the local earthquake data, a linearized discrete form relating absolution travel time residuals (d_i^{loc}) to the perturbations of the slowness (Δs_{ik}) and hypocenter location (Δh_{ij}) is expressed as:

$$d_i^{loc} = t_i^{obs} - t_i^{pred} = \sum_k \left(\frac{\partial t}{\partial s}\right)_{ik} \Delta s_{ik} + \sum_j \left(\frac{\partial t}{\partial h}\right)_{ij} \Delta h_{ij} = [G_s^{loc} \quad G_h^{loc}] \begin{bmatrix} m_s \\ m_h \end{bmatrix} \quad \text{(S1)}$$

where t_i^{obs} and t_i^{pred} are observed and predicted travel times for one ray *i*. *k* and *j* then represent the dimension of slowness model (m_s) and source parameters (m_h), through partial derivatives G_s^{loc} and G_h.

For the teleseismic data, we follow the ACH method (Aki et al., 1977) to relate the perturbations of the slowness (Δs_{ik}) to relative travel time residuals (d_i^{tel}) in a linear form as

$$d_i^{tel} = \left(t_i^{obs} - \overline{t_i^{obs}}\right) - \left(t_i^{pred} - \overline{t_i^{pred}}\right)$$

$$= \left(\sum_k \left(\frac{\partial t}{\partial s}\right)_{ik} - \sum_{i=1}^{N} \sum_k \left(\frac{\partial t}{\partial s}\right)_{ik} / N\right) \Delta s_{ik} + \sum_{j=4} \left(\frac{\partial t}{\partial h}\right)_{ij} \Delta h_{ij}$$

$$= [G_s^{loc} \quad G_h^{tel}] \begin{bmatrix} m_s \\ m_h \end{bmatrix} \quad \text{(S2)}$$

where $\overline{t_i^{obs}}$ and $\overline{t_i^{pred}}$ are the mean of travel times from one teleseismic event to a group of *N* stations including the ray *i*, so the partial derivatives, G_s^{tel} are also demeaned accordingly. For source parameter in teleseismic cases only the origin time term is included as the events are outside the model space. Ray tracing for both local earthquake and teleseismic events is calculated by the 3-D spherical pseudo-bending method[43,44] to trace and update at each iteration for a nonlinear approximation. Combing the two, a matrix form for the joint inversion is as

$$\begin{bmatrix} G_s^{loc} & G_h^{loc} \\ G_s^{tel} & G_h^{tel} \end{bmatrix} \begin{bmatrix} m_s \\ m_h \end{bmatrix} = \begin{bmatrix} d^{loc} \\ d^{tel} \end{bmatrix} \qquad \text{(S3)}$$

The velocity model is then converted from the derived slowness model in the end. With regularization imposed (e.g. damping and smoothing), the matrix then becomes as

$$\begin{bmatrix} WG \\ \lambda I \\ \varphi L \end{bmatrix} m = \begin{bmatrix} Wd \\ 0 \\ 0 \end{bmatrix} \qquad \text{(S4)}$$

where *G*, *m*, and *d* represent the equation S3. W is a weighting matrix with picking weighting placed on diagonal elements. I and L are the unit matrix and the finite-difference Laplacian operators constraining the magnitude and roughness variance over the model space. With different damping (λ) and smoothing (φ) coefficients, a series

of tests is typically performed for building a L curve of residual root-mean-square (RMS) vs. model norm and seeking the optimal values around the maximum cruvature of the curve. The λ=20 and φ=10 are chosen based on the testing curves (red dots, Figure S3).

2. Checkerboard tests and resolution index

The checkerboard tests are conducted for model resolution assessment. In the tests, we input a checkerboard-like model with -5% and 5% variations in *P*-wave velocity (V_P), interchanging with three nodes horizontally and with five nodes vertically. The same damping (20) and smoothing factors (10) as for the actual data inversion are used for checkerboard test inversion. Results show generally good recovery down to 70 km (Figure S5). Below 70 km, the well recovered areas mainly around the subducting Philippine Sea plate because of subduction seismicity.

Based on the results of the checkerboard test, we translated the model recovery level into a resolvability index, *R*, which is defined as[45]

$$R = \frac{\sum_{i=i-n}^{i+n}\sum_{j=j-n}^{j+n}\sum_{k=k-1}^{k+1}\left(Vt_{i,j,k}+Vr_{i,j,k}\right)^2}{2\sum_{i=i-n}^{i+n}\sum_{j=j-n}^{j+n}\sum_{k=k-1}^{k+1}\left(Vt_{i,j,k}^2+Vr_{i,j,k}^2\right)} \quad \text{(S5)}$$

where *Vt* are the true velocities (i.e., from a known synthetic model) and *Vr* are the recovered velocities at nodes denoted by indices *i, j, k* in 3-D space. This resolvability factor is then operated over a defined range by a desired number of nodes, *n*. A larger value of *n* produces a smoother map and vice versa. We chose *n* = 5 in this case, which is slightly larger than the perturbation wavelength (*n* = 3) in the checkerboard test and generally produces good results. *R* ranges from 0 to 1, in which *R* = 1 represents the velocity anomaly is 100% recovered (nodes with significant ray crossing), *R* = 0.5 indicates 0% recovered (nodes with no rays crossing), and *R* = 0 denotes a velocity that is −100% perturbed (unstable inversion nodes). The corresponding *R* index and the derivative weighted sum (DWS)[46] map at different depths are also shown in Figure S4. The DWS at each velocity model node can be viewed as a proxy for the ray density. Compared to the recovery of the checkerboard tests, *R* = 0.6 is considered a reasonable lower bound for a resolvable area[29]. *R* is therefore used as an index to mask the areas where values smaller than 0.6 as in Figure 2-4.

3. Characteristic-model tests

Geometry test. Two possible anomaly geometries are tested to evaluate the smearing effect for L1 slow anomaly (Figure S7): one is vertically-elongated (8-20 km) as the actual inversion images (Figure 3) and the other one is vertically-concentrated (12-16 km) for a more sill-like structure. The vertically-elongated scenario performs a better fit (Figure S7c and S7h) than the vertically-concentrated scenario (Figure S7e and S7j).

Since the smearing effect is not sever for both scenarios, the vertically-elongated geometry of the L1 slow anomaly should be robust.

Data noise test. We also test the scenario with data noise in Figure S8. Based on the residual RMS of actual inversion (Figure S4), the random noise with 0.12-s standard deviation is added into synthetic data for inversion. The results show very similar images to the noise-free scenario and indicate that at least at the depth range of interest the L1 slow anomaly is robustly constrained when a small amount of data noise is present.

Initial model test. The effect of different initial model is examined in Figure S9. We show that while the 3-D velocity model of Huang et al. (2014)[23] is used as the initial model for actual data inversion (Figure 2, 3, and 4), the results starting from the 1-D initial model (average from the 3-D model) also reach similar images. This indicates that the L1 slow anomaly is not controlled or biased by existing structures in the 3-D initial model.

Anomaly magnitude test. Lastly, since the regularization imposed in the tomographic inversion often damps the magnitude of velocity perturbations, we perform a series of tests with different input Vp reductions for the L1 slow anomaly to assess their actual velocity anomaly magnitude. Figure S10 shows the testing results of -16%, -19%, and -22% Vp reductions where the -19% produce closest magnitude of velocity anomalies in actual inversion (Figure 3).

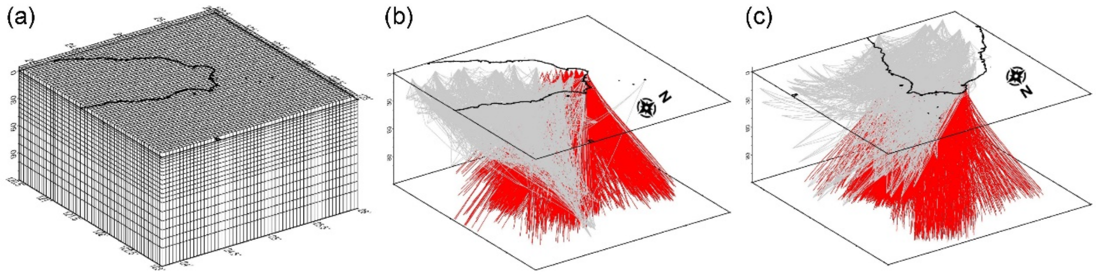

Figure S1. 3-D perspective of the (a) model parameterization and (b and c) ray path distribution of local (gray lines) and teleseismic events (red lines) from two different angles.

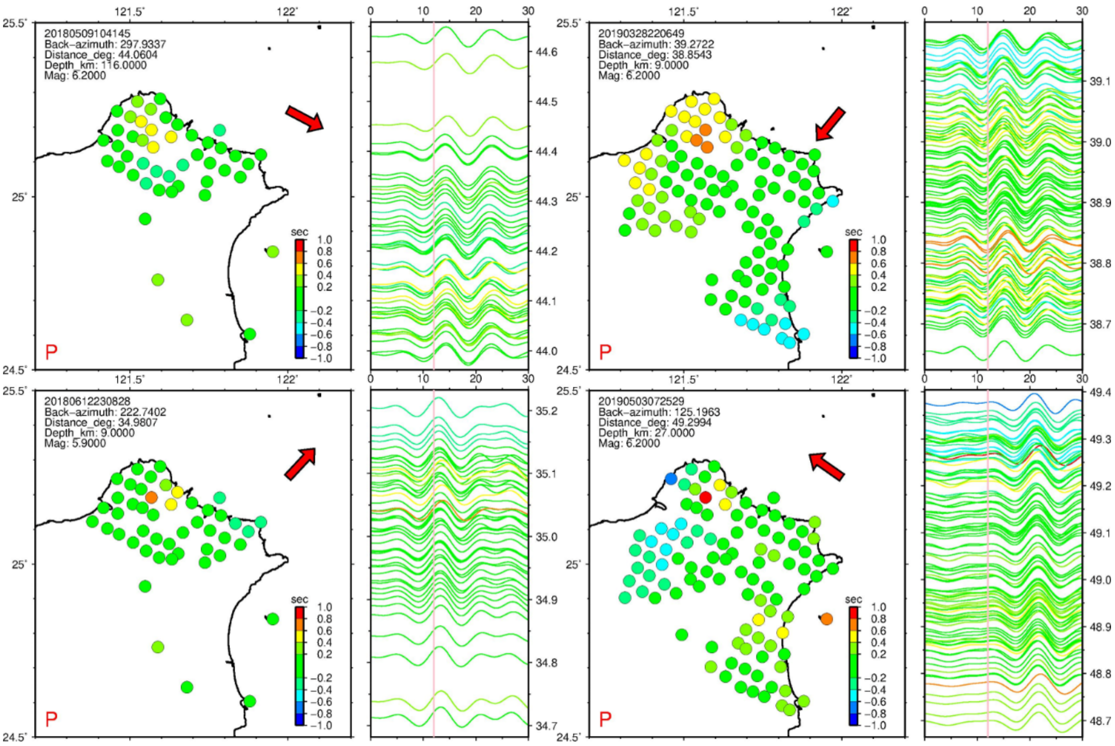

Figure S2. Measurement examples of P-wave relative arrival times from different event azimuths in four quadrants. The colored dots on the map show the relative times at stations measured from waveform cross correlations on the right column. All the waveforms are aligned with the predicted arrival times (red vertical lines) from the 1D global model *ak135*. The red arrows on the map indicate the event azimuths. The earthquake information is shown on the upper left corner of each map.

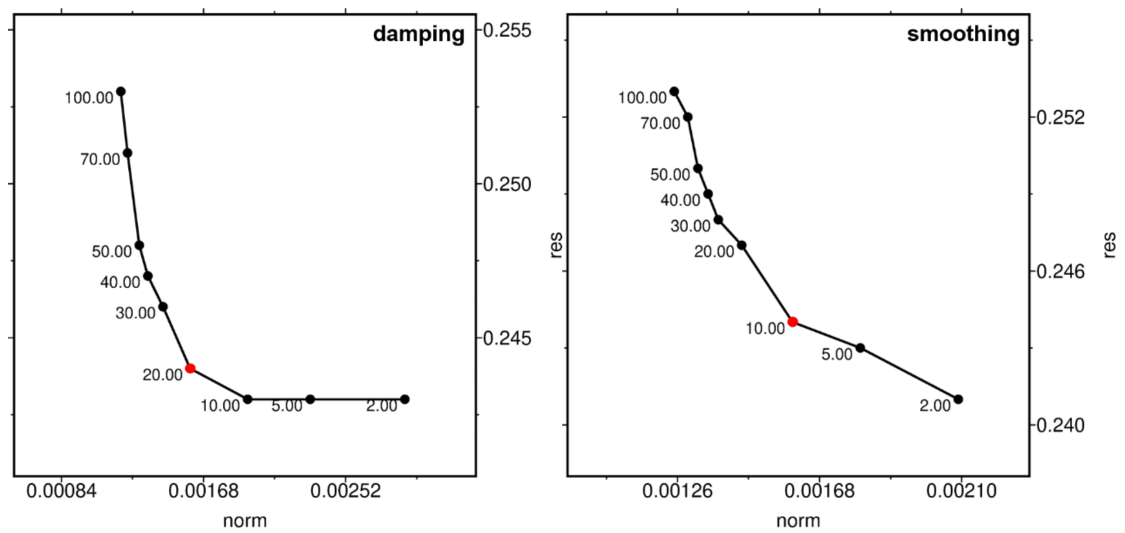

Figure S3. Trade-off tests for damping and smoothing parameter selection. Red dots are selected values for the inversion.

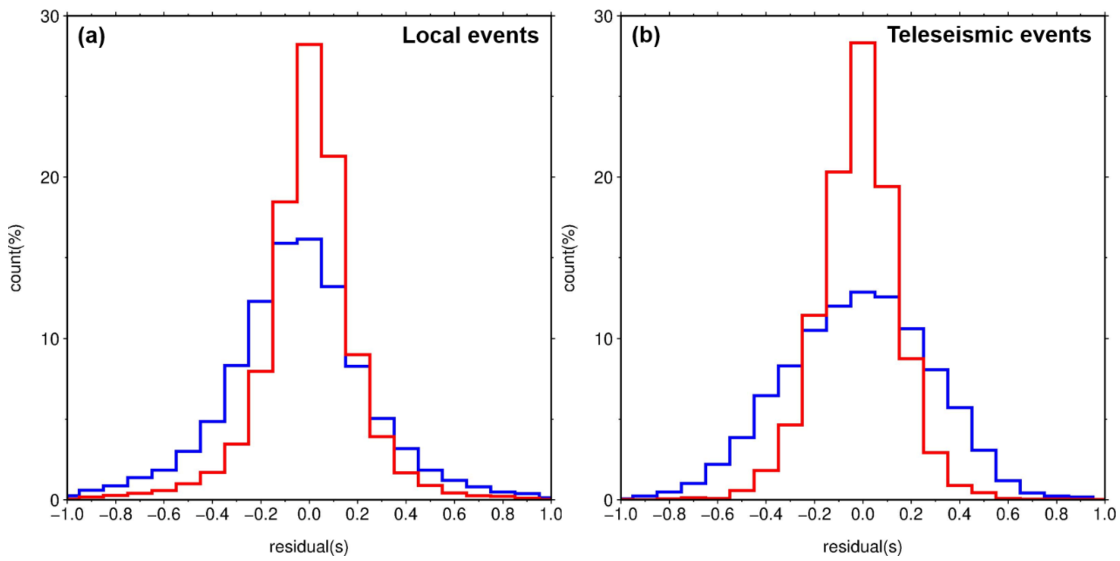

Figure S4. Distribution of (a) local earthquake and (b) teleseismic earthquake residuals before and after the joint inversion in blue and red histograms, respectively.

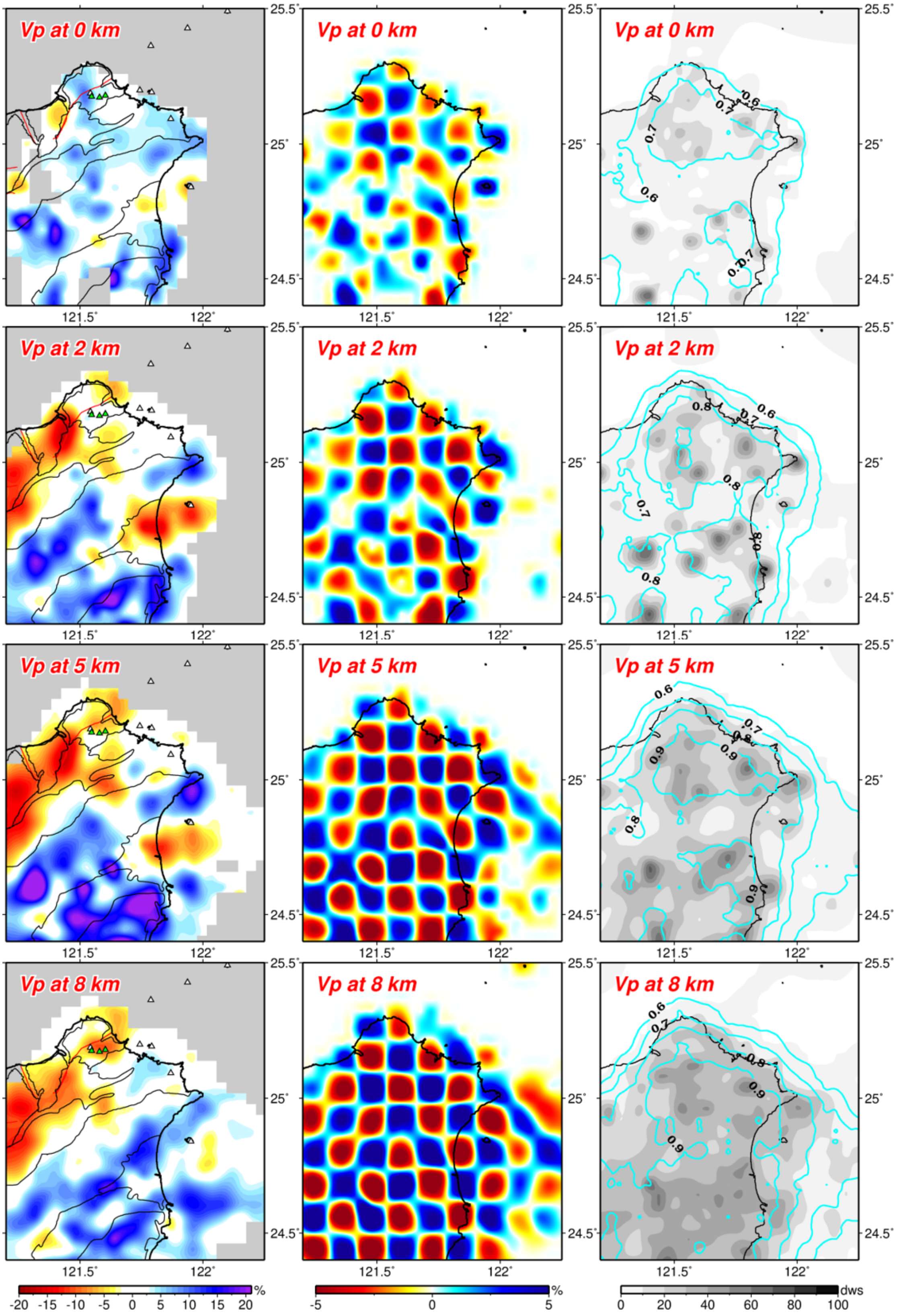
Vp at 0 km
Vp at 2 km
Vp at 5 km
Vp at 8 km
25.5°
25°
24.5°
121.5°
122°
0.6
0.7
0.8
0.9
%
-20 -15 -10 -5 0 5 10 15 20
-5 0 5
dws
0 20 40 60 80 100

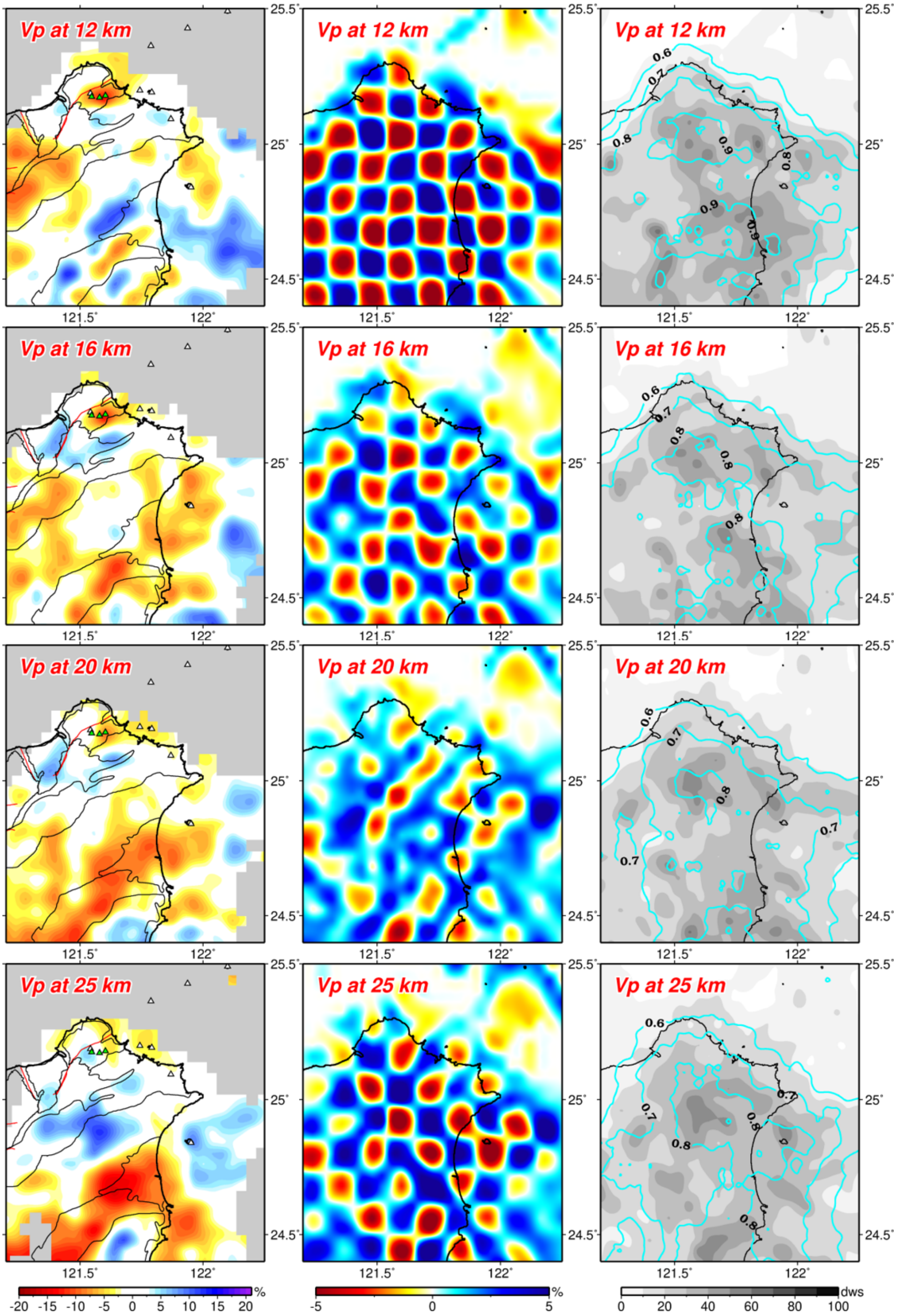

Vp at 12 km
Vp at 12 km
Vp at 12 km
Vp at 16 km
Vp at 16 km
Vp at 16 km
Vp at 20 km
Vp at 20 km
Vp at 20 km
Vp at 25 km
Vp at 25 km
Vp at 25 km
121.5°
122°
25.5°
25°
24.5°
%
-20 -15 -10 -5 0 5 10 15 20
%
-5 0 5
dws
0 20 40 60 80 100

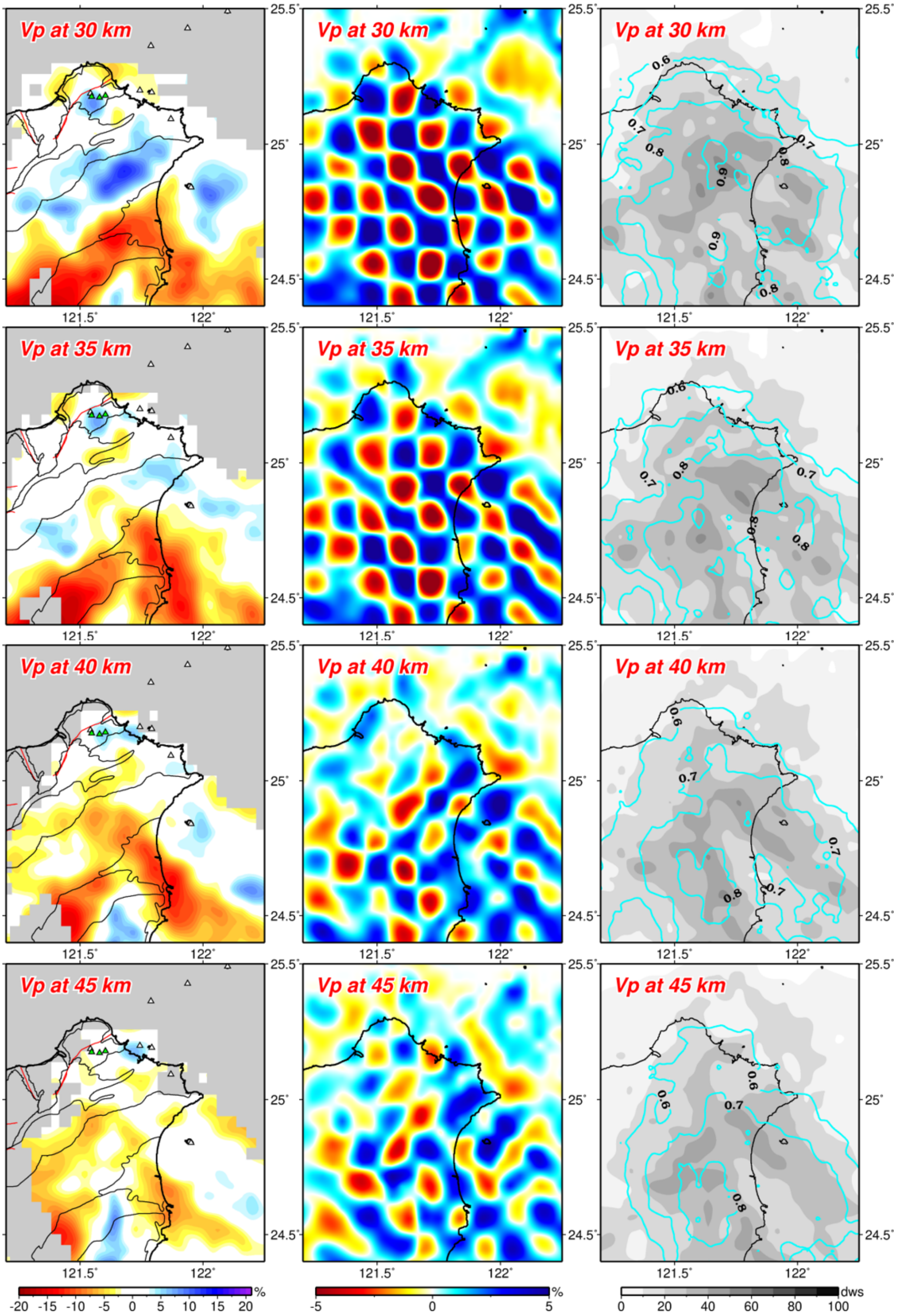

Vp at 30 km
Vp at 30 km
Vp at 30 km
Vp at 35 km
Vp at 35 km
Vp at 35 km
Vp at 40 km
Vp at 40 km
Vp at 40 km
Vp at 45 km
Vp at 45 km
Vp at 45 km
25.5°
25°
24.5°
121.5°
122°
-20 -15 -10 -5 0 5 10 15 20 %
-5 0 5 %
0 20 40 60 80 100 dws

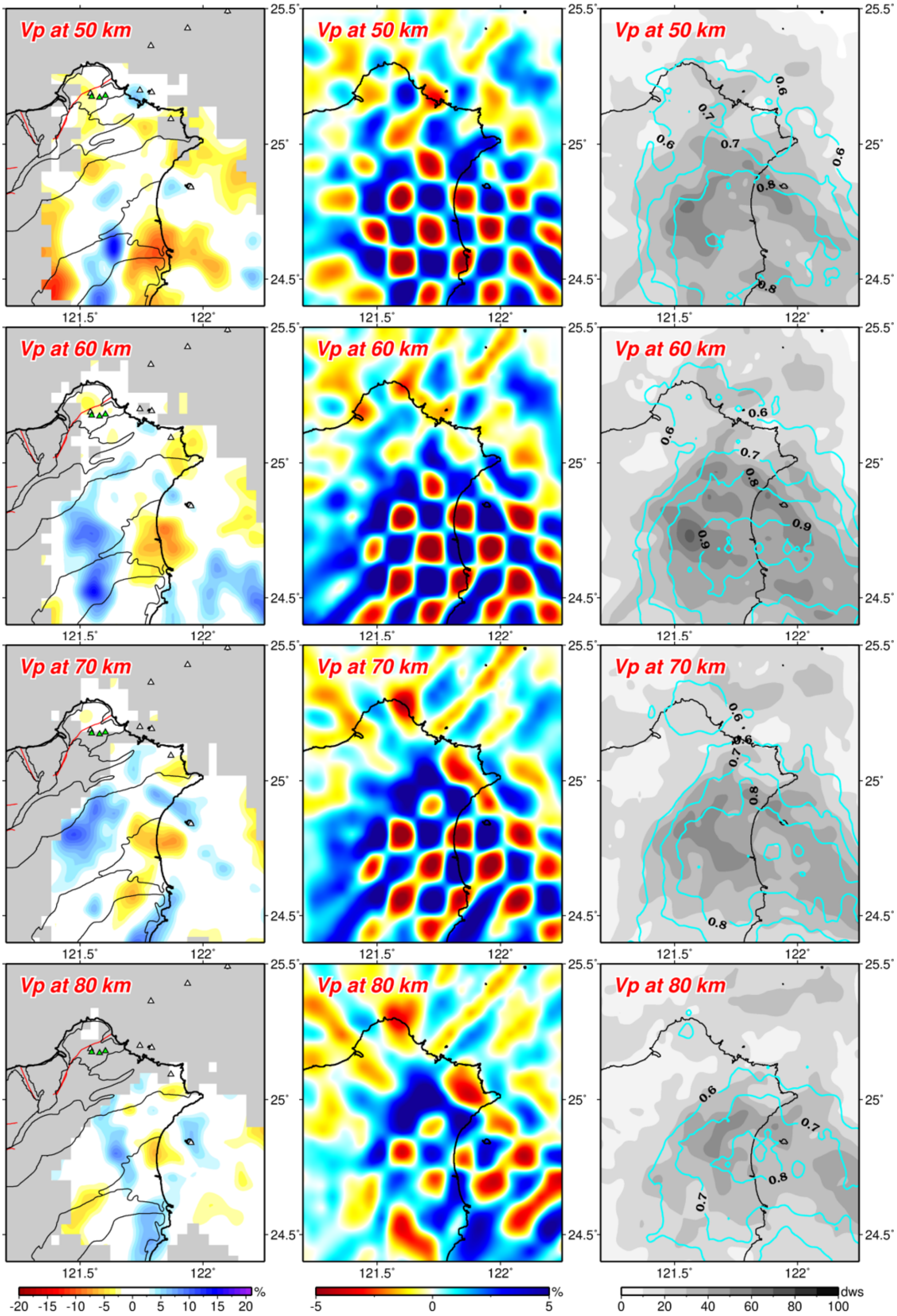
Vp at 50 km
Vp at 60 km
Vp at 70 km
Vp at 80 km
121.5°
122°
25.5°
25°
24.5°
0.6
0.7
0.8
0.9
%
-20 -15 -10 -5 0 5 10 15 20
-5 0 5
dws
0 20 40 60 80 100

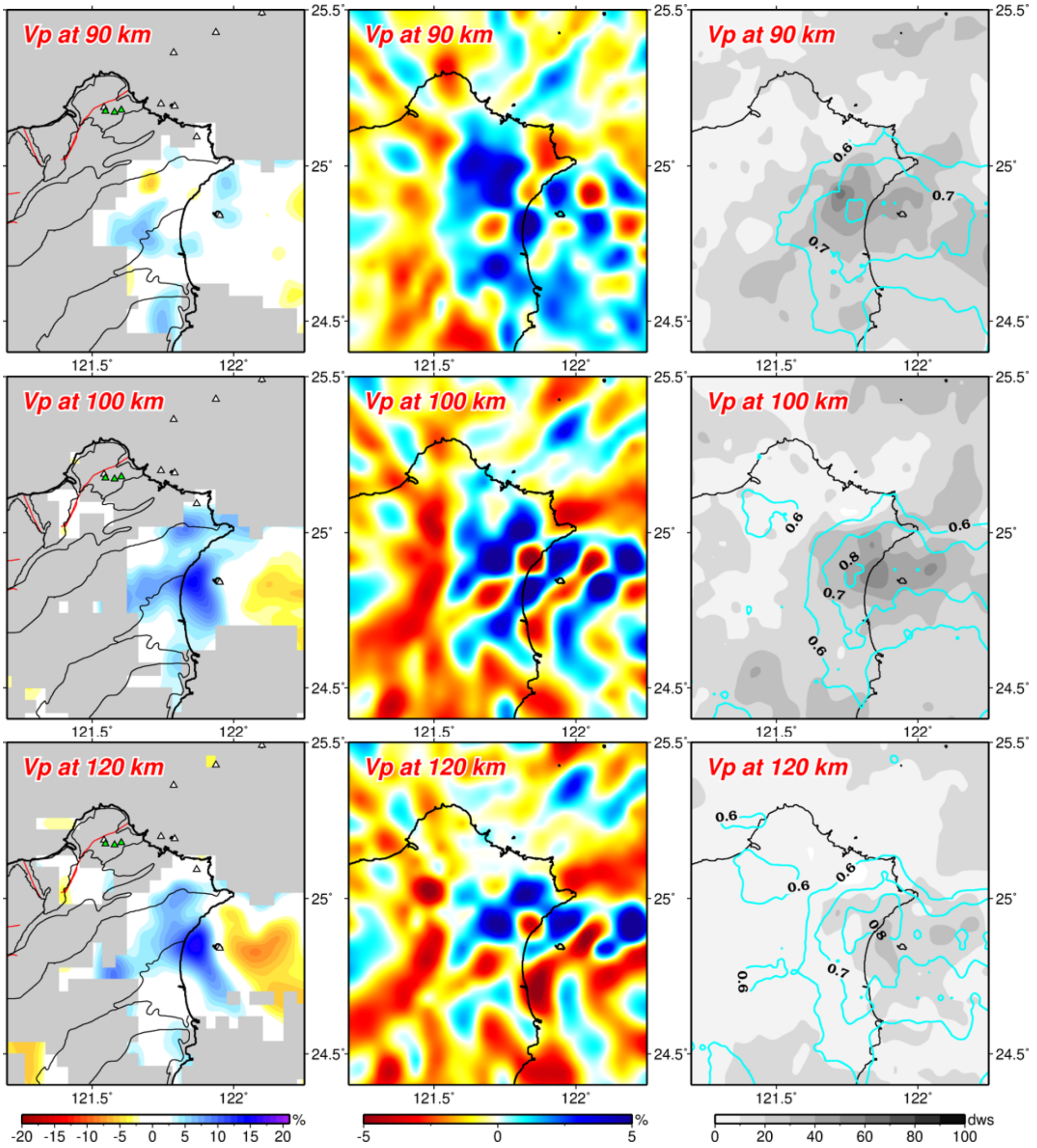

Figure S5. Tomographic results and resolution tests at different depths. From left to right are the inversion results, checkerboard tests, and resolution maps. In the resolution maps, the contours and background colors show the calculated resolvability index, R, and the derivative weighted sum, DWS. This figure is generated by GMT 4.5.18 https://www.generic-mapping-tools.org/

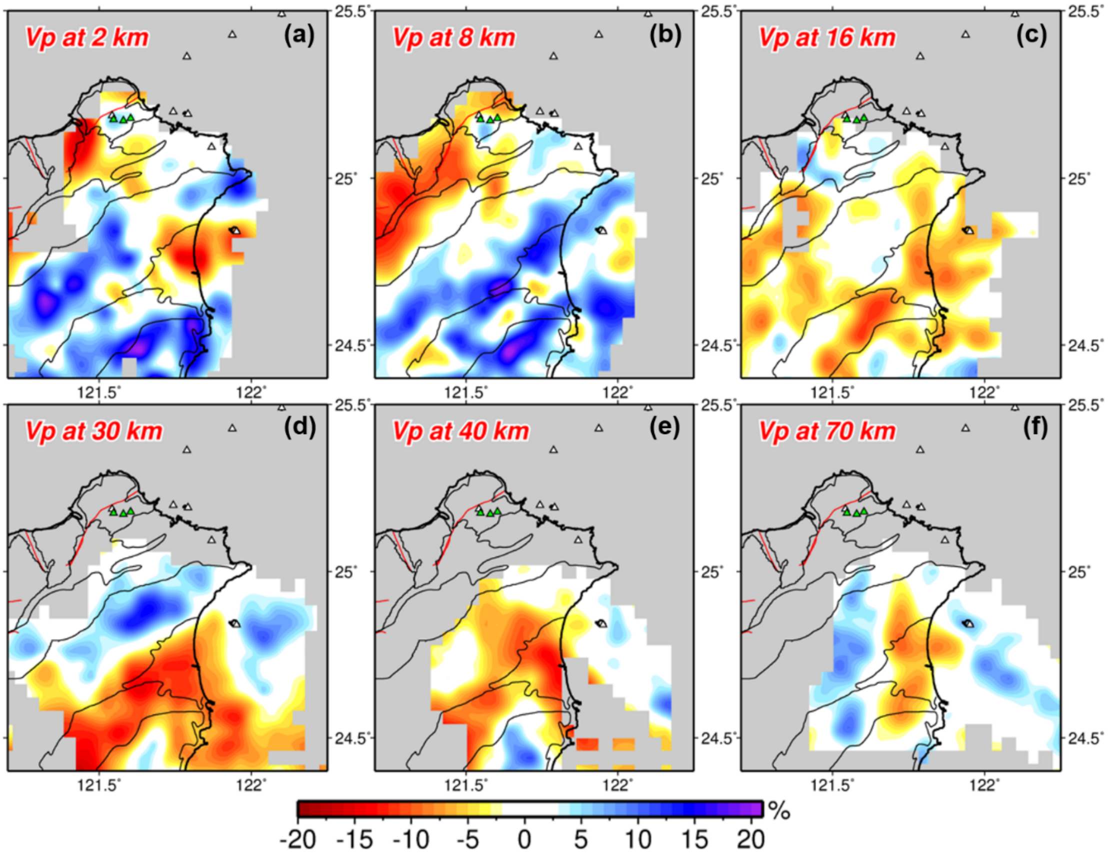

Figure S6 Model results from the inversion using only local earthquake data at different depths (a-f). Warm and cold colors represent the low and high P-wave velocities. White triangles denote the volcano locations of Northern Taiwan volcanic zone. Green triangles are main fumarole sites and craters in Tatun volcano group. This figure is generated by GMT 4.5.18 https://www.generic-mapping-tools.org/

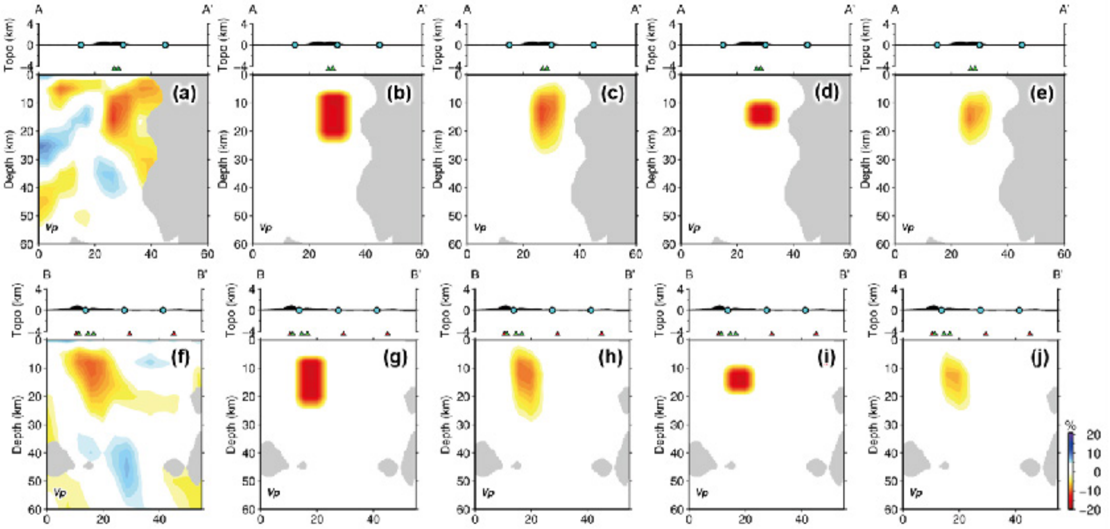

Figure S7. Characteristic-model tests for vertically-elongated geometry of L1 slow anomaly. According to the real data inversion results in two cross-sectional views (a, f), two different depth ranges of input anomalies (velocity reduction of 19%) are placed at 8-20 (b, g) and 12-16 km (d, i) for testing. The recovered images are shown in (c, h) and (e, j), respectively.

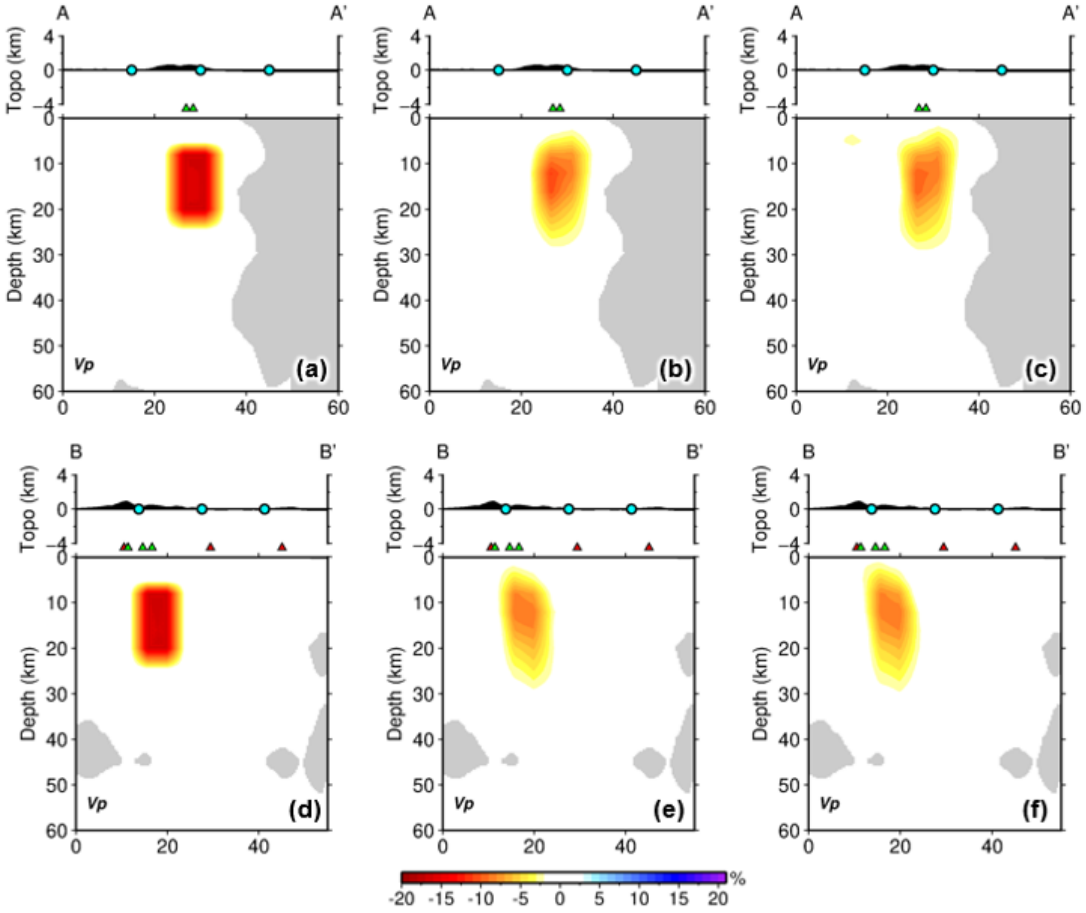

Figure S8. Data noise sensitivity tests. (a-c) are the input anomaly and the recovered images with and without data noise. (d-f) show the other cross-sectional view. According to the final residuals in the real data inversion, the random data noise with zero mean and a standard deviation of 0.12 s are added.

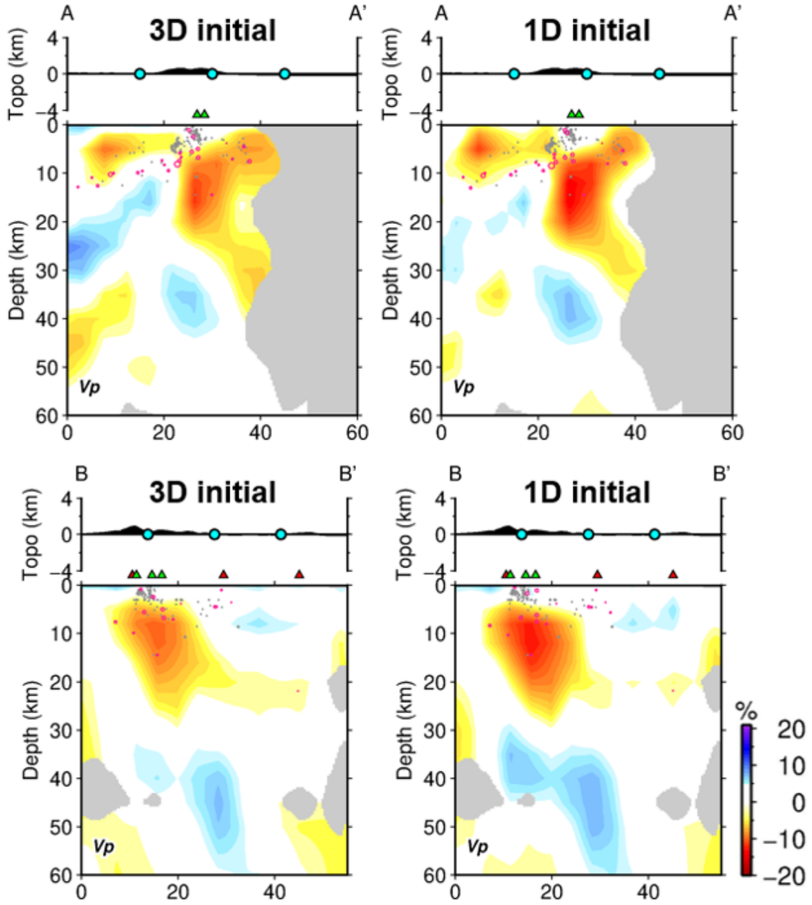

Figure S9. Comparison of the model results from the inversions using 3-D (left) and 1-D (right) initial models. The general pattern of the velocity variations are very similar while the magnitude slightly differs.

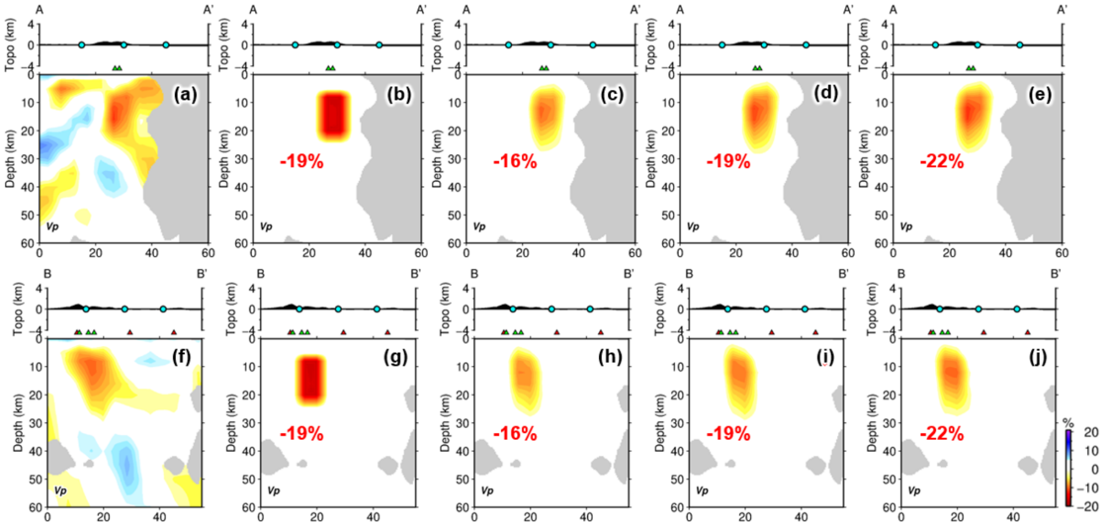

Figure S10. Characteristic-model tests for actual magnitude of L1 slow anomaly. (a, f) and (b, g) show the real data inversion results and the input anomaly geometry in two cross-sectional views. The recovered images for the -16%, -19%, and -22% input velocity anomalies are shown in (c, h), (d, i), and (e, j), respectively.

Table S1. Setting of the model grids in depth.

Grid depth (km)	Vp (km/s)
-5	3.000
0	3.847
2	4.447
5	4.969
8	5.394
12	5.812
16	6.110
20	6.383
25	6.662
30	6.997
35	7.398
40	7.556
45	7.714
50	7.871
60	7.955
70	8.039
80	8.100
90	8.162
100	8.228
120	8.347

Table S2. Physical properties of rocks and fluids used for the calculation. Superscript numbers denote the references of the chosen values listed below.

Material	Vp (km/s)	Vs (km/s)	ρ(g/cm^3)	Critical porosity
Granite	5.7[1]	3.4[1]	2.63[1]	0.35[5]
Peridotite	8.0[2]	3.7[2]	3.3[2]	0.04[6]
Andesitic melt	2.8[3]	0	2.7[4]	
Basaltic melt	2.9[3]	0	2.8[4]	
CO_2	0.675[7]	0	0.37[7]	
Water	0.82[7]	0	0.23[7]	

1 Watanabe (1994), measurements under 625°C and 0.42 GPa

2 Christensen (2004), measurements under room temperature and 1 GPa

3 Ueki and Iwamori (2016), measurements under 1000°C and 1 GPa

4 Lesher and Spera (2015), measurements under 1000°C and 1 GPa

5 van der Molen and Paterson (1979)

6 Schmeling (2006)

7 Chu et al. (2010)

References

46. Um, J., & Thurber, C. A fast algorithm for two-point seismic ray tracing. Bull. Seismol. Soc. Am. **77**, 972–986 (1987).
47. Koketsu, K., & Sekine, S. Pseudo-bending method for three-dimensional seismic ray tracing in a spherical earth with discontinuities. Geophys. J. Int. **132(2)**, 339–346 (1998).
48. Zelt, C. A. Lateral velocity resolution from three-dimensional seismic refraction data. Geophys. J. Int. **135**, 1101–1112 (1998).
49. Toomey, D. R., & Foulger, G. R. Tomographic inversion of local earthquake data from the Hengill-Grensdalur central volcano complex, Iceland. J. Geophys. Res. **94**, 17,497–17,510, doi:10.1029/JB094iB12p17497 (1989).
